# Predicting the Onset of Acute Encephalopathy With Biphasic Seizures and Late Reduced Diffusion by Using Early Laboratory Data

**DOI:** 10.3389/fneur.2021.730535

**Published:** 2021-11-01

**Authors:** Masanori Maeda, Tohru Okanishi, Yosuke Miyamoto, Takuya Hayashida, Tatsuya Kawaguchi, Sotaro Kanai, Yoshiaki Saito, Yoshihiro Maegaki

**Affiliations:** ^1^Division of Child Neurology, Department of Brain and Neurosciences, Faculty of Medicine, Tottori University, Yonago, Japan; ^2^Department of Pediatrics, Wakayama Medical University, Wakayama, Japan; ^3^Department of Pediatrics, Kyoto Prefectural University of Medicine, Kyoto, Japan; ^4^Department of Pediatrics, Nagasaki University, Nagasaki, Japan; ^5^Division of Child Neurology, Saiseikai Yokohama City Tobu Hospital, Yokohama, Japan

**Keywords:** acute encephalopathy, biphasic seizures, diffusion, children, early diagnosis, laboratory data

## Abstract

**Background:** Acute encephalopathy with biphasic seizures and late reduced diffusion (AESD) often causes various neurological sequelae, necessitating early and objective differentiation of AESD from a febrile seizure (FS). Therefore, we developed a scoring system that predicts AESD onset using only early laboratory data.

**Methods:** We selected patients with AESD or FS admitted to the Tottori University Hospital between November 2005 and September 2020 and collected laboratory data from onset to discharge in patients with FS and from onset to the second neurological events in patients with AESD.

**Results:** We identified 18 patients with AESD and 181 patients with FS. In comparison with patients with FS, patients with AESD showed statistically significant increases in ammonia (NH3), blood sugar (BS), and serum creatinine (Cr) levels, and the white blood cell (WBC) count, and a significant decrease in pH at <3 h from onset. We set the cut-off values and adjusted the weight of each of these parameters based on data obtained <3 h from onset and proposed a scoring system for predicting AESD. This system showed 91% sensitivity and 94% specificity for distinguishing AESD from FS. These accuracies were only slightly improved by the addition of information related to consciousness and seizure duration (sensitivity, 91%; specificity, 96%).

**Conclusion:** NH3, BS, and Cr levels, WBC count, and pH were significantly different between patients with AESD and patients with FS at <3 h from seizure onset. This scoring system using these data may enable the prediction of AESD onset for patients under sedation or without precise clinical information.

## Introduction

Acute encephalopathy with biphasic seizures and late reduced diffusion (AESD) represents one of the clinico-radiological parainfectious neurological syndromes, classified as a whole as acute parainfectious encephalopathies. AESD affects infants and toddlers in East Asia, particularly in Japan, and can lead to mild to severe neurological sequelae (1). The biphasic course of AESD is characteristic as compared with other acute parainfectious encephalopathies, namely febrile infection-related epilepsy syndrome (FIRES)/acute encephalitis with refractory, repetitive partial seizures (AERRPS), mild encephalopathy with a reversible splenial lesion (MERS), acute necrotizing encephalopathy (ANE), and hemorrhagic shock and encephalopathy syndrome (HSES) (1). The initial symptom of AESD is a convulsive seizure with parainfectious fever, and it is often challenging to distinguish this encephalopathy from febrile seizure (FS) in the early phase. On the other hand, other encephalopathies are clinically differentiated from FS: FIRES/AERRPS is characterized by the super-refractory status epilepticus predominantly constituted by repetitive focal onset seizures; the main symptom of MERS at the onset is delirious behavior; ANE/HSES can be differentiated based on its rapid aggravation of consciousness followed by shock state (1).

A nationwide survey in Japan from 2014 to 2017 revealed that AESD constitutes 34% of acute infectious encephalopathies, and ~100 children develop this syndrome annually (2). AESD is characterized clinically by the onset of febrile convulsive seizures commonly lasting longer than 30 min (early seizure) and the emergence of focal motor seizures (late seizures) or consciousness impairment on the fourth to 6th day of the early seizure. Variable levels of consciousness impairment have been observed between the early and late seizures. Restricted diffusion in the cerebral subcortical white matter on diffusion-weighted magnetic resonance imaging (DWI) (the so-called “bright tree” appearance) is usually detected around the 7th day of the disease course. Neurological sequelae, including intellectual disability, paralysis, and epilepsy, are seen in more than 60% of patients; this ratio did not change from the previous nationwide survey in Japan conducted during 2007–2010 (2).

Some studies have indicated that early interventions [therapeutic hypothermia (3), vitamin B1, vitamin B6, and L-carnitine administration (4), and dextromethorphan treatment (5)] may improve the outcomes of AESD, and it is clinically important to distinguish AESD from FS in the early phase after the onset of initial convulsive seizure. Some patients with FS experience prolonged seizures and impaired consciousness for several hours. However, some patients with AESD develop early seizures shorter than 30 min (median: 4 min; approximately 20% of patients) (6) or completely recover their consciousness after early seizures (~20% of patients) (7).

Previous studies have reported two scoring systems that evaluated clinical and laboratory findings in patients with AESD or FS presenting with seizures longer than 30 min for early prediction of AESD (8, 9). Tada et al. (8) selected seven variables, namely, age, consciousness level 12–24 h after onset of the seizure, duration of the seizure, mechanical ventilation, and blood sugar (BS), serum creatinine (Cr), and aspartate aminotransferase (AST) levels. Yokochi et al. (9) selected six variables, including time until waking, BS, Cr, ammonia (NH3), and alanine aminotransferase (ALT) levels, and pH. In these studies, the laboratory data during the first convulsion phase were used, although the exact time of sampling or subsequent evolution of the data were not evaluated. Therefore, the optimal sampling time for predicting AESD has not been clarified. Other concerns about the scoring systems include the profound influence of anticonvulsive drug usage or observer subjectivity on the scoring of items such as the duration of the seizure, consciousness, and mechanical ventilation. Thus, for more objective evaluations to differentiate AESD from FS, it may be better to use only laboratory data considering the time of sampling.

In this study, we aimed to clarify the relationship between the sampling time and the changes in laboratory data and to provide a scoring system for AESD/FS differentiation using only laboratory data obtained at seizure onset.

## Materials and Methods

### Patient Criteria and Clinical Information

We used medical records to select patients aged under 16 years who developed convulsive seizures complicated by fevers and were admitted to Tottori University Hospital between November 2005 and September 2020. We then identified patients with final diagnoses of AESD or FS. In this study, we defined FS based on the following criteria: (1) convulsive seizures with fever (>38°C); (2) onset without the appearance of other causes of acute symptomatic seizures, including central nervous system infection or acute encephalopathy; (3) recovery of consciousness within 24 h after seizure termination; and (4) no sequelae after the seizure. We defined AESD based on the following criteria: (1) onset of febrile convulsive seizure (early seizure) followed by recovery of consciousness to some extent; (2) secondary convulsive seizures (late seizures) or impaired consciousness on the fourth to 6th day of the early seizure; and (3) restricted diffusion in the cerebral subcortical white matter on DWI (bright tree appearance). Cases with unclear secondary convulsions due to anticonvulsive drugs for refractory seizures were included if the bright tree appearance sign was confirmed on DWI. We excluded children with seizure-related neurological disorders, including congenital central nervous anomalies, refractory epilepsies, and known metabolic or genetic disorders causing epilepsy.

The following clinical information was recorded: age (months), sex, perinatal status, neurological background disorders (intellectual disability, developmental disorders, and history of febrile or afebrile seizures), duration of the seizure in FS and early seizure in AESD, the lowest consciousness level, assessed by the Glasgow coma scale (GCS), at 12–24 h after the first seizure, and interventions including anticonvulsant treatments, intubation/artificial ventilation and intravenous hydration, that can affect the initial blood sampling data.

### Laboratory Data and MRI Findings

Laboratory data, including NH3, BS, Cr, AST, ALT, lactate dehydrogenase (LDH), creatine kinase (CK), and C-reactive protein (CRP) levels, white blood cell (WBC) and platelet (Plt) counts, and pH, were available for this study. We collected all laboratory data from onset to discharge in patients with FS and from onset to secondary neurological events in patients with AESD. We also recorded the sampling time from the seizure onset for each datum.

We reviewed brain DWI scans and identified bright tree appearance signs as high-intensity lesions in the cerebral subcortical white matter during the fourth to 14th day after the early seizure.

### Statistical Analyses

We compared clinical characteristics and laboratory data between the patients with AESD and FS by using Fisher's exact test for categorical data and the Mann–Whitney *U* test for continuous or ordinal data. Next, we employed multivariate regression analyses to evaluate which covariates of sampling time from the seizure onset (h), diagnosis of AESD or FS, and duration of the first seizure affected each laboratory datum. Based on the findings of these initial analyses, we categorized the sampling time from seizure onset into three periods: <3, 3, to <24, and ≥24 h. Then, we compared the laboratory data for each sampling time category between patients with AESD and FS by using the Mann–Whitney *U*-test. Statistical analyses were performed using BellCurve for Excel (BellCurve for Excel 3.21, Social Survey Research Information, Tokyo, Japan). Statistical significance was set at *p* < 0.05.

### Prediction Scoring for AESD Using Early Laboratory Data

To establish prediction scoring in the early period of onset, we used the data obtained <3 h of onset, which could be used for early differentiation. Using the receiver operating characteristic (ROC) curve, we determined cutoff values for laboratory data that showed significant differences between patients with AESD and FS. Next, using univariate regression analysis, we confirmed that the laboratory data binarized by the cutoff values significantly correlated with the diagnosis of AESD. For the prediction scoring system, we entered only the data showing significant correlations. These variables were scored as 1 or 2 according to the size of each regression coefficient (RC). We proposed a laboratory prediction score for AESD and determined its sensitivity and specificity using the ROC curve and Youden index.

### AESD Prediction Scoring System Including Clinical Data

To evaluate the accuracy of the scoring system using only laboratory data, we developed another scoring system that also included seizure duration and GCS data. The cut-off values for seizure duration and GCS score were also determined and scored using the respective RCs. We did not include information on age and sex in this scoring system because patients with AESD and patients with FS showed no significant differences for these parameters (see subsection 3.1.). We also did not include information on background neurological disorders because the corresponding data were extremely limited.

### Ethical Approval

This study was approved by the local ethics committee of Tottori University Hospital (approval number: 20A214). Informed consent was obtained in the form of opt-outs posted on the website and in the hospital.

## Results

### Patient Characteristics

We identified 18 patients with AESD and 181 patients with FS. The median age at onset was 23 months in patients with AESD and 18.5 months in patients with FS (Table 1). Seven (39%) patients with AESD and 108 (60%) patients with FS were males. Developmental delays, neonatal asphyxia, extremely low birth weight, or afebrile seizure/epilepsy prior to onset were significantly more frequent in the AESD group than the FS group (*p* = 0.0011, *p* = 0.0056, *p* < 0.001, respectively), whereas the incidence of developmental disorders was not significantly different between the two groups. The seizure duration in patients with AESD was significantly longer than that in patients with FS (median duration: 50 min and 20 min, respectively; *p* = 0.0018). The GCS scores were lower in the AESD group than in the FS group (median scores: 12 and 15, respectively; *p* < 0.001). The ratios of the interventions were not significantly different between the two groups.

**Table 1:** Clinical information.

**Characteristics**	**AESD** **(*n* = 18)**	**FS** **(*n* = 181)**	***p*** **-value**
**Age (months)**			
Median (range)	23 (9–70)	18.5 (5–101)	0.280
**Sex**, ***n*** **(%)**			
Male	7 (38.9)	108 (59.7)	0.132
**Background disorders**, ***n*** **(%)**			
Total	7 (38.9)	11 (6.1)	<0.001^**^
Developmental delay	5 (27.8)	6 (3.3)	0.0011^**^
Developmental disorder	0 (0)	3 (1.7)	1.000
Neonatal asphyxia/ELBW	3 (16.7)	2 (1.1)	0.0056^**^
Afebrile seizure/epilepsy	5 (27.8)	2 (1.1)	< 0.001^**^
**Seizure duration (min)**			
Median (range)	50 (3–103)	20 (0.2–120)	0.0018^**^
**Glasgow coma scale score**, ***n*** **(%)**			< 0.001^**^
15	3 (16.7)	154 (85.1)	
13–14	5 (27.8)	26 (14.4)	
9–12	6 (33.3)	1 (0.6)	
3–8	4 (22.2)	0 (0)	
**Interventions before the blood sampling**, ***n*** **(%)**			
Anticonvulsant treatments	0 (0)	0 (0)	1.000
Intubation and artifical ventilation	0 (0)	0 (0)	1.000
Intravenous hydration	1 (5.6)	15 (8.3)	1.000

*We used Fisher's exact test and Mann–Whitney U test appropriately.*

** *p < 0.01*.

### Laboratory Data

#### The Laboratory Data in Patients With AESD and FS

**Figure 1:**
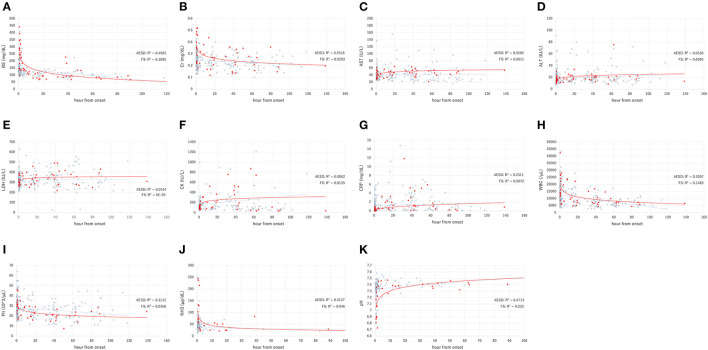
The laboratory data values, including the levels of blood sugar (BS) **(A)**, serum creatinine (Cr) **(B)**, aspartate aminotransferase (AST) **(C)**, alanine aminotransferase (ALT) **(D)**, lactate dehydrogenase (LDH) **(E)**, creatine kinase (CK) **(F)**, C-reactive protein (CRP) **(G)**, white blood cell (WBC) count **(H)**, platelet count (Plt) **(I)**, ammonia (NH3) level **(J)**, and pH **(K)**, in acute encephalopathy with biphasic seizures and late reduced diffusion (AESD) (filled triangles), and febrile seizure (FS) (open circles). The X and Y axes show the hours from onset and the value, respectively. The solid line and dotted line indicate the fitted curves for AESD and FS, respectively.

Figure 1 and Table 2 show the values of laboratory data, including BS, Cr, AST, ALT, LDH, CK, CRP, and NH3 levels; WBC and Plt counts; and pH and the range of sampling time after seizure onset in patients with AESD and FS.

**Table 2:** The values of laboratory data and the range of sampling time from seizure onset in AESD and FS patients.

		**AESD** **(*n* = 18)**	**FS** **(*n* = 181)**
BS	Sample size	53	238
	Value [mg/dL, median (range)]	130 (68–433)	124 (71–409)
	Time range (h from onset)	0.6–139	0.1–118
Cr	Sample size	44	279
	Value [mg/dL, median (range)]	0.27 (0.12–0.52)	0.23 (0.1–0.43)
	Time range (h from onset)	0.6–139	0.1–118
AST	Sample size	45	287
	Value [U/L, median (range)]	47 (22–65)	37 (16–156)
	Time range (h from onset)	0.6–139	0.1–118
ALT	Sample size	45	287
	Value [IU/L, median (range)]	18 (9–76)	17 (7–91)
	Time range (h from onset)	0.6–139	0.1–118
LDH	Sample size	45	278
	Value [IU/L, median (range)]	331 (223–495)	316 (26–626)
	Time range (h from onset)	0.6–139	0.1–118
CK	Sample size	41	246
	Value [IU/L, median (range)]	134 (33–877)	114 (37–3,973)
	Time range (h from onset)	0.6–139	0.1–118
CRP	Sample size	45	283
	Value [mg/dL, median (range)]	0.67 (0.02–11.9)	0.61 (0.01–14.8)
	Time range (h from onset)	0.6–139	0.1–118
WBC	Sample size	43	285
	Value [/μL, median (range)]	9,800 (4,500–42,500)	9,600 (2,500–43,200)
	Time range (h from onset)	0.7–139	0.1–118
Plt	Sample size	42	283
	Value [ ×10^3^/μL, median (range)]	23.2 (7.6–46)	25.8 (10–64.1)
	Time range (h from onset)	0.7–139	0.1–118
NH3	Sample size	23	165
	Value [μg/dL, median (range)]	57 (23–245)	44 (11–248)
	Time range (h from onset)	0.7–89	0.2–83
pH	Sample size	30	177
	Value [median (range)]	7.36 (6.73–7.48)	7.37 (6.79–7.58)
	Time range (h from onset)	0.6–89	0.1–90

#### Associations Between Clinical Factors and Laboratory Data

To clarify the factors that influenced changes in laboratory data, we used multivariate regression analysis using the sampling time from seizure onset and diagnosis of AESD or FS as the explanatory variables. Furthermore, the seizure duration, which showed significant differences between patients with AESD and FS in this study (Table 1), was also included as an explanatory variable since this factor possibly affected the changes in laboratory data. Table 3 presents the results of each analysis. A longer duration of seizures was significantly associated with the BS (RC: 0.222; *p* < 0.001) and NH3 (RC: 0.206; *p* = 0.005) levels and the pH (RC: −0.331; *p* < 0.001). A longer sampling time from seizure onset was significantly associated with the BS (RC: −0.4; *p* < 0.001), Cr (RC: −0.215; *p* < 0.001), and NH3 (RC: −0.207; *p* = 0.004) levels, WBC (RC: −0.403; *p* < 0.001) and Plt (RC: −0.209; *p* < 0.001) counts, and pH (RC: 0.292; *p* < 0.001). AESD diagnosis was significantly associated with BS (RC: 0.138; *p* = 0.021), Cr (RC: 0.239; *p* < 0.001), AST (RC: 0.134; *p* = 0.023), and NH3 (RC: 0.232; *p* = 0.002) levels, the WBC count (RC: 0.12; *p* = 0.027), and pH (RC: −0.187; *p* = 0.009).

**Table 3:** Multivariate analyses for variables associating with each laboratory datum.

**Response variables**	**Explanatory variables**	**Regression coefficient**	***p*** **-value**
BS	Duration of the seizures	0.222	<0.001^**^
	Sampling time from the seizure onset	−0.4	<0.001^**^
	AESD or FS	0.138	0.021^*^
Cr	Duration of the seizures	0.012	0.839
	Sampling time from the seizure onset	−0.215	<0.001^**^
	AESD or FS	0.239	<0.001^**^
AST	Duration of the seizures	0.057	0.332
	Sampling time from the seizure onset	0.056	0.308
	AESD or FS	0.134	0.023^*^
ALT	Duration of the seizures	0.041	0.488
	Sampling time from the seizure onset	0.056	0.306
	AESD or FS	0.113	0.056
LDH	Duration of the seizures	0.078	0.198
	Sampling time from the seizure onset	0.022	0.697
	AESD or FS	0.036	0.554
CK	Duration of the seizures	0.019	0.768
	Sampling time from the seizure onset	0.109	0.069
	AESD or FS	0.009	0.885
CRP	Duration of the seizures	−0.007	0.903
	Sampling time from the seizure onset	0.097	0.082
	AESD or FS	0.044	0.467
WBC	Duration of the seizures	0.061	0.259
	Sampling time from the seizure onset	−0.403	<0.001^**^
	AESD or FS	0.12	0.027^*^
Plt	Duration of the seizures	0.016	0.779
	Sampling time from the seizure onset	−0.209	<0.001^**^
	AESD or FS	−0.066	0.258
NH3	Duration of the seizures	0.206	0.005^**^
	Sampling time from the seizure onset	−0.207	0.004^**^
	AESD or FS	0.232	0.002^**^
pH	Duration of the seizures	−0.331	<0.001^**^
	Sampling time from the seizure onset	0.292	<0.001^**^
	AESD or FS	−0.187	0.009^**^

*We used multivariate regression analyses.*

* *p < 0.05,*

** *p < 0.01*.

#### Comparisons of the Laboratory Data Between Patients With AESD and FS at Each Sampling Time Period

**Figure 2:**
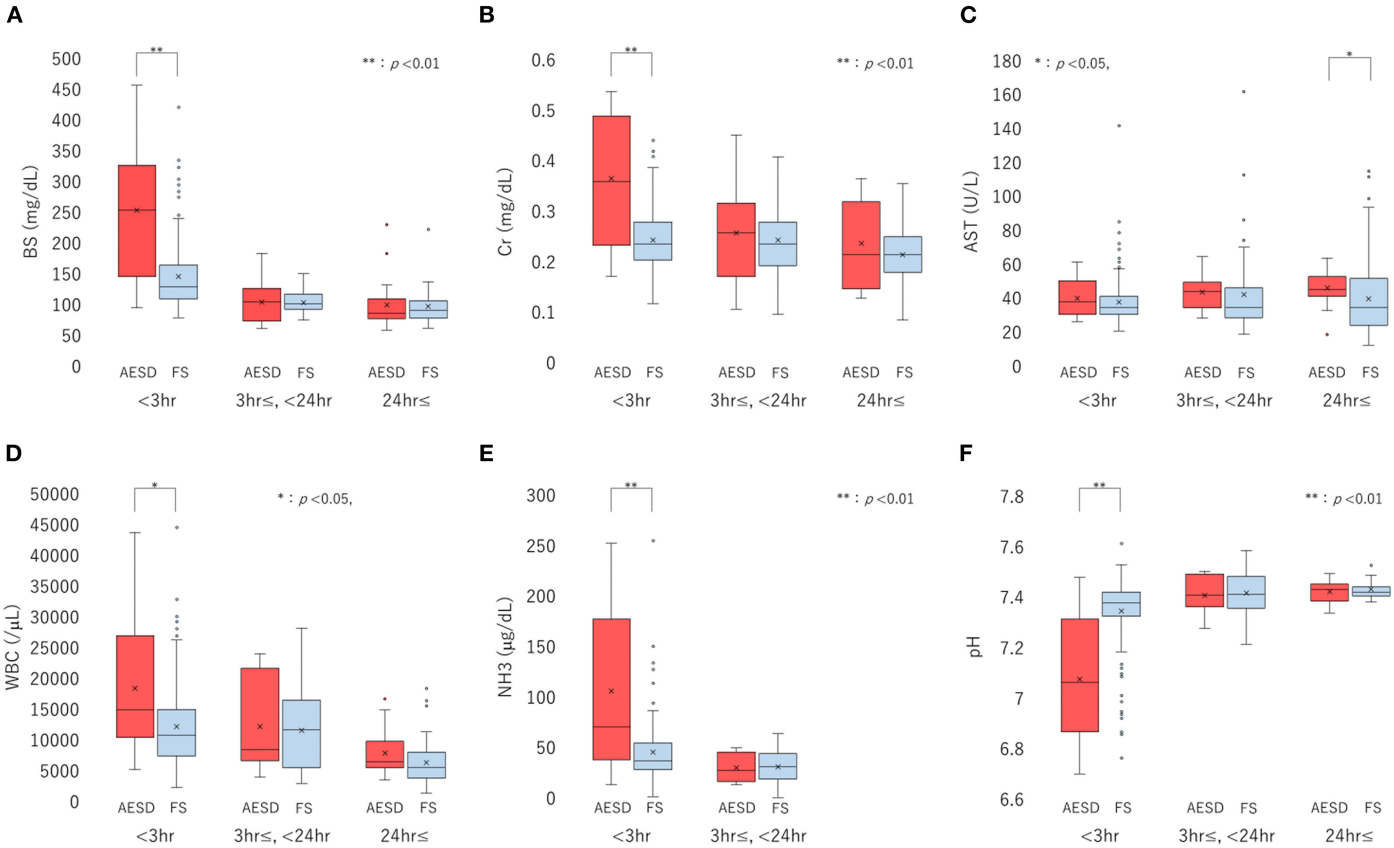
Boxplots of laboratory data, including the levels of blood sugar (BS) **(A)**, serum creatinine (Cr) **(B)**, and aspartate aminotransferase (AST) **(C)**, white blood cell (WBC) count **(D)**, ammonia (NH3) level **(E)**, and pH **(F)**, divided into three sampling-time periods from onset: <3 h, 3 h to <24 h, and ≥24 h. Each box, horizontal line in box, and cross mark indicates the interquartile range (IQR), median, and mean, respectively. Whiskers denote the range between 1.5 times the IQR upper from the third quartile and lower from the first quartile, and circles indicate outliers. The distributions of laboratory data are compared between biphasic seizures and late reduced diffusion (acute encephalopathy with biphasic seizures and late reduced diffusion [AESD]) and febrile seizure (FS) at each period, using the Mann-Whitney U test. BS **(A)** and Cr **(B)** levels, WBC count **(D)**, and NH3 level **(E)** are significantly higher in AESD than FS at <3 h, and pH **(F)** was significantly lower in AESD than FS at <3 h. AESD patients showed a significant increase in AST level **(C)** at ≥24 h.

We divided the sampling time from the seizure onset into three periods: <3 h, 3 h– <24 h, and ≥24 h, and compared the laboratory data between patients with AESD patients and patients with FS for each period. The six laboratory parameters showing significant associations between the values and sampling times in Section 3.2.2, namely, BS, Cr, AST, and NH3 levels, WBC count, and pH (Table 3), were selected for comparison. In patients with AESD, the number of datasets ranged from 13 to 19 at <3 h, from 7 to 17 between 3 and 24 h, and from 8 to 19 for ≥24 h. In patients with FS, the number ranged from 142 to 167 at <3 h, from 20 to 44 between 3 and 24 h, and from 12 to 83 for ≥24 h. Since the data for NH3 levels during the ≥24 h period was limited in both patients with AESD and FS, the analysis of NH3 levels during the third period was not available (Figure 2).

In comparison of patients with FS, patients with AESD showed statistically significant increases in BS [median (range), 251 (102–443) in AESD vs. 134 (87–409) in FS; *p* < 0.001] and Cr [median (range), 0.36 (0.18–0.52) vs 0.24 (0.13–0.43); *p* < 0.001] levels, WBC count [median (range), 15,300 (6,100–42,500) vs. 11,400 (3,400–43,200); *p* = 0.026], and NH3 level [median (range), 76 (23–245) vs. 45 (12–248), *p* = 0.008] at <3 h (Figure 2). Patients with AESD showed a statistically significant decrease in pH [median (range), 7.07 (6.73–7.45) vs. 7.36 (6.79–7.58), *p* < 0.001] at <3 h (Table 4).

**Table 4:** The differences of values between AESD and FS patients at each period of sampling time.

	**Sampling time from the seizure onset**	**AESD patients median (range)**		**FS patients median (range)**	***p*** **-value**
BS	<3 h	251 (102–443) (*n =* 19)	>	134 (87–409) (*n =* 167)	<0.001^**^
	3 h ≤, <24 h	111 (70–185) (*n =* 17)		108 (83–154) (*n =* 35)	0.8682
	24 h ≤	93 (68–229) (*n =* 17)		98.5 (71–222) (*n =* 36)	0.3962
Cr	<3 h	0.355 (0.18–0.52) (*n =* 14)	>	0.24 (0.13–0.43) (*n =* 158)	<0.001^**^
	3 h ≤, <24 h	0.26 (0.12–0.44) (*n =* 12)		0.24 (0.11–0.40) (*n =* 43)	0.7129
	24 h ≤	0.22 (0.14–0.36) (*n =* 18)		0.22 (0.10–0.35) (*n =* 78)	0.4079
AST	<3 h	40 (29–62) (*n =* 14)		37 (24–137) (*n =* 160)	0.3042
	3 h ≤, <24 h	45.5 (31–65) (*n =* 12)		37 (22–156) (*n =* 44)	0.1056
	24 h ≤	47 (22–64) (*n =* 19)	>	37 (16–112) (*n =* 83)	0.0162^*^
WBC	<3 h	15,300 (6,100–42,500) (*n =* 14)	>	11,400 (3,400–43,200) (*n =* 161)	0.0263^*^
	3 h ≤, <24 h	9,200 (5,000–23,900) (*n =* 12)		12,250 (4,000–27,800) (*n =* 42)	0.8028
	24 h ≤	7,400 (4,500–17,000) (*n =* 17)		6,500 (2,500–18,600) (*n =* 82)	0.174
NH3	<3 h	76 (23–245) (*n =* 13)	>	45 (12–248) (*n =* 143)	0.0082^**^
	3 h ≤, <24 h	36 (23–57) (*n =* 8)		39.5 (11–70) (*n =* 20)	0.7986
	24 h ≤	-		-	-
pH	<3 h	7.07 (6.73–7.45) (*n =* 14)	<	7.36 (6.79–7.58) (*n =* 142)	<0.001^**^
	3 h ≤, <24 h	7.39 (7.27–7.48) (*n =* 7)		7.39 (7.21–7.55) (*n =* 23)	0.9218
	24 h ≤	7.41 (7.32–7.47) (*n =* 9)		7.40 (7.36–7.50) (*n =* 12)	1

*We used the Mann–Whitney U test;*

* *p < 0.05,*

** *p < 0.01*.

Patients with AESD showed a significant increase in the AST level at ≥24 h [median (range), 47 (22–64) vs. 37 (16–112), *p* = 0.016]. Significant differences were not observed between patients with AESD and patients with FS in the data obtained between 3 and 24 h (Table 4).

### Prediction Scoring Systems for AESD and Their Accuracies

#### Prediction Scoring Using Only Laboratory Data

Using the ROC curve, we determined the following cutoff values for BS, Cr, and NH3 levels, WBC count, and pH at <3 h to distinguish AESD from FS: BS ≥ 220 mg/dL; Cr ≥ 0.35 mg/dL; WBC ≥ 25000/μL; NH3 ≥ 75 μg/dL; and pH ≤ 7.25. Univariate regression analysis confirmed that each of the cutoff values was significantly correlated with AESD, including BS level (RC: 0.562; *p* < 0.001), Cr level (RC: 0.479; *p* < 0.001), WBC count (RC: 0.270; *p* = 0.0014), NH3 level (RC: 0.396; *p* < 0.001), and pH (RC: 0.394; *p* < 0.001) (Table 5).

**Table 5:** Cutoff points and univariate analyses for each laboratory datum which was significantly different between AESD and FS patients at the period of <3 h.

**Response variables**	**Explanatory variables**	**Regression coefficient (ratio between WBC)**	***p*** **-value**
AESD or FS	BS ≥ 220	0.562 (2.08)	<0.001^**^
	Cr ≥ 0.35	0.479 (1.77)	<0.001^**^
	WBC ≥ 25,000	0.270 (1)	0.0014^**^
	NH3 ≥ 75	0.396 (1.47)	<0.001^**^
	pH ≤ 7.25	0.394 (1.46)	<0.001^**^

*We used univariate regression analyses.*

** *p < 0.01*.

**Figure 3:**
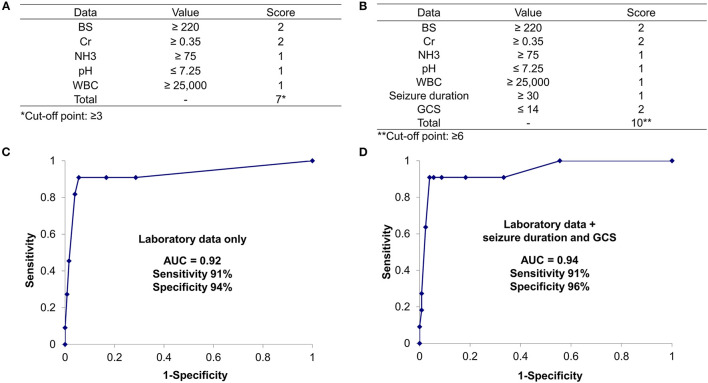
The prediction scores for acute encephalopathy with biphasic seizures and late reduced diffusion (AESD) using only laboratory data, including blood sugar (BS) level (≥ 220 mg/dL, scored as 2 points), serum creatinine (Cr) level (≥0.35 mg/dL, 2 points), white blood count (WBC) (≥25,000/μL, 1 point), ammonia (NH3) level (≥75 μg/dL, 1 point), and pH (≤7.25, 1 point) **(A)**. The prediction scores for AESD using laboratory data, seizure duration (≥ 30 min, 1 point), and Glasgow coma scale (GCS) score (≤14, 2 points) **(B)**. Receiver–operator characteristic curve for the scoring model using only laboratory data **(C)** and laboratory data with seizure duration and GCS score **(D)**. Using the cutoff point at a total score ≥ 3, the sensitivity and the specificity are 91 and 94%, respectively, for the scoring model with only laboratory data **(C)**. In the scoring model using laboratory data with seizure duration and GCS score, using the cutoff point at a total score ≥6, the sensitivity and the specificity are 91 and 96%, respectively **(D)**. AUC, area under the curve.

BS and Cr levels were scored 2 while WBC count, NH3 level, and pH were scored 1 above each cutoff value for the AESD prediction scoring system, which was assigned based on the ratios of RC values for the WBC count to those for the other parameters (BS: 2.08; Cr, 1.77; NH3, 1.47; pH, 1.46) (Table 5). We derived the laboratory prediction score for AESD in patients with AESD and patients with FS for whom all predictors were available (11 patients with AESD and 126 patients with FS) (Figure 3). The cutoff point for predicting AESD determined from the ROC curve was a total score of ≥3. The sensitivity and specificity of this scoring system were 91% and 94%, respectively.

#### Prediction Scoring Using Laboratory Data With Clinical Information

We set the cut-off values for seizure duration at 30 min and GCS score at 14. Univariate regression analyses showed significant correlations with the onset of AESD, both for seizure duration (RC: 0.257; *p* = 0.0024) and GCS score (RC: 0.466; *p* < 0.001). Using the values of RCs, seizure duration ≥ 30 min was scored as 1, and GCS score ≤ 14 was scored as 2.

Using the clinical information added to the laboratory data, the cutoff point for predicting AESD was a total score ≥ 6. This scoring system showed 91% sensitivity and 96% specificity, which were slightly different from the values obtained using only laboratory data (Figure 3).

## Discussion

### Time Courses of Laboratory Data in AESD and FS

We clarified that the BS and Cr levels, WBC count, NH3 level, and pH were significantly different between patients with AESD and patients with FS when obtained <3 h from the seizure onset and were not significantly different at periods ≥3 h. Tada et al. (8) and Yokochi et al. (9) had reported scoring systems for predicting AESD using initial laboratory data from seizure onset, although the timing of blood sampling was not evaluated in those studies. The present study recommends the use of laboratory data only at early sampling within 3 h from the onset for prediction. However, the AST level in patients with AESD was significantly higher than that in patients with FS only after 24 h. Although AST and ALT levels were included in the predictive scoring systems in previous studies, it would not be appropriate to use these data to predict the onset of AESD at 24 h of seizure onset. According to our analyses of the time course of the laboratory data, we observed that the BS and Cr levels, WBC count, NH3 level, and pH may be suitable for early prediction of AESD.

### Prediction Scores for AESD and Advantages of Using Only Laboratory Data

In our study, the scoring systems using laboratory data only and laboratory data with consciousness scores and seizure duration showed nearly equivalent accuracies. Additionally, the accuracy of our scoring system using only laboratory data was comparable with those of the scoring systems used in previous studies (sensitivity = 89% and specificity = 90% in Tada et al.'s report (8) and sensitivity = 93% and specificity = 91% in Yokochi et al.'s report (9), in which clinical information of patient age, consciousness level, and seizure duration were regarded as critical for predicting AESD. The consciousness level after the seizure and the duration of the seizure may be important predictors since these were significantly different between patients with AESD and patients with FS in our study as well. However, the level of consciousness is often influenced by the use of anticonvulsive drugs for ceasing early seizures. Moreover, the evaluation of seizure duration by caregivers and physicians is sometimes subjective. The actual seizure duration is sometimes not evaluable, for example, in AESD cases with non-convulsive seizures (10). We considered that the present scoring system using only laboratory data offered the advantage of objective evaluation.

### Possible Mechanisms Underlying the Changes in Blood Laboratory Data in AESD

The univariate analysis evaluated the correlations of each laboratory parameter with the diagnosis of AESD, and the highest RC was obtained for the BS level. Early seizures in AESD may induce prominent activation of the paraventricular nucleus followed by elevation of cortisol, catecholamine, and proinflammatory cytokine levels, the development of stress-induced hyperglycemia, as well as an increase in the WBC count (11, 12). The Cr level showed the second highest RC for the diagnosis of AESD in this study. Although Cr levels generally reflect acute renal injury, an increase in the Cr level could reflect energy failure in the central nervous system (8, 13, 14). Creatine and phosphocreatine are the sources of Cr and play important roles in buffering ATP in high-energy-requiring cells such as neurons. Increased ATP synthesis results in a decrease in phosphocreatine and an increase in Cr levels in these cells. Creatine/phosphocreatine peaks decreased in subcortical white matter in magnetic resonance spectroscopy in patients with AESD with mild to severe sequelae within 7 days of onset (15). Early seizures in AESD require more ATP synthesis than FS, whereas elevated Cr levels in the brain might be released into the systemic circulation because of the disrupted blood brain barrier in AESD (16). Prolonged seizures decrease pH and increase BS and NH3 levels (17, 18); these data might be replaceable with seizure duration as a scoring item.

### Limitations

This study had several limitations. First, the number of patients with AESD in our study was smaller than that in previous studies on prediction of AESD onset, and the reported accuracy of the scoring system may have been higher than that in reality. Further investigation in a larger cohort is necessary to validate the accuracy of our prediction score. Second, the laboratory data could have been influenced by interventions such as administration of anticonvulsants, artificial ventilation, and intravenous hydration.

### Conclusion

The development of AESD was predicted using our scoring system, which included only laboratory data obtained < 3 h from seizure onset.

## Data Availability Statement

The original contributions presented in the study are included in the article/supplementary material, further inquiries can be directed to the corresponding author.

## Ethics Statement

The studies involving human participants were reviewed and approved by Tottori University Hospital. Written informed consent to participate in this study was provided by the participants' legal guardian/next of kin.

## Author Contributions

MM, TO, SK, and YMa conceptualized and designed the study, carried out the analyses, and drafted the initial manuscript. YMi, TK, TH, and YS collected patient data for this study. All authors approved the final manuscript as submitted and agree to be accountable for all aspects of the work.

## Funding

This research was supported by Grant-in-Aid for Policy Research for Intractable Diseases, H30-Nanchi-Ippan-007, from the National Institute of Public Health, Japan.

## Conflict of Interest

The authors declare that the research was conducted in the absence of any commercial or financial relationships that could be construed as a potential conflict of interest.

## Publisher's Note

All claims expressed in this article are solely those of the authors and do not necessarily represent those of their affiliated organizations, or those of the publisher, the editors and the reviewers. Any product that may be evaluated in this article, or claim that may be made by its manufacturer, is not guaranteed or endorsed by the publisher.
